# Analyzing large-scale spiking neural data with HRLAnalysis^™^

**DOI:** 10.3389/fninf.2014.00017

**Published:** 2014-03-05

**Authors:** Corey M. Thibeault, Michael J. O'Brien, Narayan Srinivasa

**Affiliations:** Center for Neural and Emergent Systems, Information and Systems Sciences Laboratory, HRL Laboratories LLC.Malibu, CA, USA

**Keywords:** python, spiking neural data analysis, high-performance computing, spike train analysis, data sharing

## Abstract

The additional capabilities provided by high-performance neural simulation environments and modern computing hardware has allowed for the modeling of increasingly larger spiking neural networks. This is important for exploring more anatomically detailed networks but the corresponding accumulation in data can make analyzing the results of these simulations difficult. This is further compounded by the fact that many existing analysis packages were not developed with large spiking data sets in mind. Presented here is a software suite developed to not only process the increased amount of spike-train data in a reasonable amount of time, but also provide a user friendly Python interface. We describe the design considerations, implementation and features of the HRLAnalysis^™^ suite. In addition, performance benchmarks demonstrating the speedup of this design compared to a published Python implementation are also presented. The result is a high-performance analysis toolkit that is not only usable and readily extensible, but also straightforward to interface with existing Python modules.

## 1. Introduction

Large-scale neural simulations have become an increasingly important tool in computational neuroscience. Although the methods behind these simulations may be different they all result in an extraordinary amount of simulated data. Whether it is the inclusion of highly detailed biophysical models (Markram, 2006), huge numbers of point neurons (Izhikevich and Edelman, 2008; Ananthanarayanan et al., 2009), or nervous system spanning functional anatomy (Eliasmith et al., 2012), the data deluge is a concern. In addition, the relatively low cost of high-performance computing systems and the recent popularity of neuromorphic hardware promises to continue this trend toward larger, more detailed models. And this problem is not exclusive to neural simulations. One of the underlying goals of the recently announced BRAIN initiative^1^ is to develop tools capable of capturing the activity of at least one million neurons—an effort that will greatly improve the state of the art but also result in tremendous amounts of data.

From a software engineering perspective, this data deluge can be approached in different ways. Using strongly-typed compiled languages, such as C, offers high-performance, but can sacrifice flexibility and extensibility. Languages popular in neuroscience, such as MATLAB or Python provide a relatively simple interface but can result in poor performance for many large-scale problems. In this paper we present, HRLAnalysis^™^, a software suite that aims to alleviate these concerns when processing spiking data.

Developed as part of the DARPA SyNAPSE program (Cruz-Albrecht et al., 2012; DARPA, 2012; Srinivasa and Cruz-Albrecht, 2012), the HRLAnalysis^™^ package was initially created as an implementation of off-line analysis and visualization of spiking and network data for use with HRLSim^™^ (Minkovich et al., 2013)—although as described below, the design does not unnecessarily restrict its use to only those data formats. One of the primary results of a neural simulation are recordings of the spiking activity—action potential event times—of each of the neurons in the network. Spike-train analysis is useful for providing insight into the structure and function of a neural network or region of the nervous system. For instance the information encoded about a stimulus can be extracted using a number of different spike and rate based codes derived from spike trains (Dayan et al., 2001; Ince et al., 2009; Quiroga and Panzeri, 2009; Crumiller et al., 2011). In addition, the synchronization in the spiking of neurons may indicate a pathological condition or this can used as a measure of neuronal coding (Kreuz et al., 2013). Similarly, correlated firings can reveal information about motor behavior, attention, and external stimulus (de la Rocha et al., 2007). HRLAnalysis^™^ provides a way to efficiently calculate many of these useful metrics on a large number of spike trains. As a general tool, the scope of HRLAnalysis^™^ is limited to spike-train analysis. However, as we demonstrate, its design does allow for integration with existing tools that can complement the functions provided by HRLAnalysis^™^.

To balance efficiency with usability, the extraction—reading spike data from disk or converting other formats into spiking data—and analysis of the data is performed in C++ and the plotting and any further manipulation required by the user is handled in Python through the Boost package^2^. With this, users get the performance benefits of C++ but also the feature rich and syntactically simple interface of Python. In addition, users can take advantage of the extensive libraries available to Python for further manipulation and visualization of the results.

## 2. Methods

### 2.1. Introductory example

**Figure 1:**
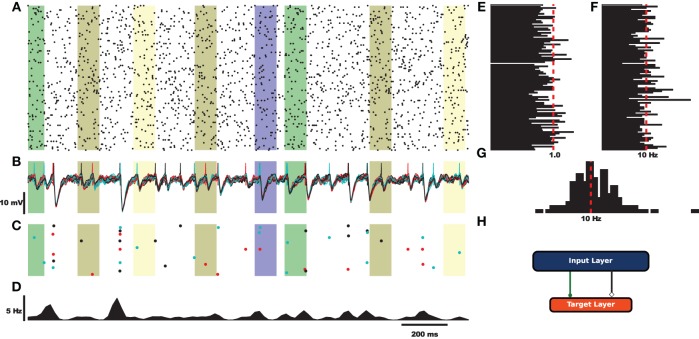
**Example analysis of simulated data using a derived voltage input class**. For this example the rectangular bars highlight regions where different signals are injected into the network. **(A)** Raster plot of 100 cells selected from the input layer. **(B)** Voltage traces of the target layer neurons. **(C)** Raster plot of the target layer. **(D)** Fire rate of the target output neurons calculated using a Gaussian window function. **(E)** Coefficient of variation for 100 cells of the input layer. **(F)** Average rate for the 100 sample cells. **(G)** Spike count histogram for the input neurons. **(H)** Fully connected feed-forward network.

Before exploring the design of HRLAnalysis^™^ in detail consider the example in Figure 1. This gives a qualitative illustration of the results for a simulated two-layer network using a class that reads in HRLSim^™^ voltage files (included with the codebase). The plot was created using the Matplotlib library^3^. Listing 1 is the relevant code required to create this plot. Note that this is incomplete and assumes that the subplots have been initialized on Line 4.

**Listing 1:**
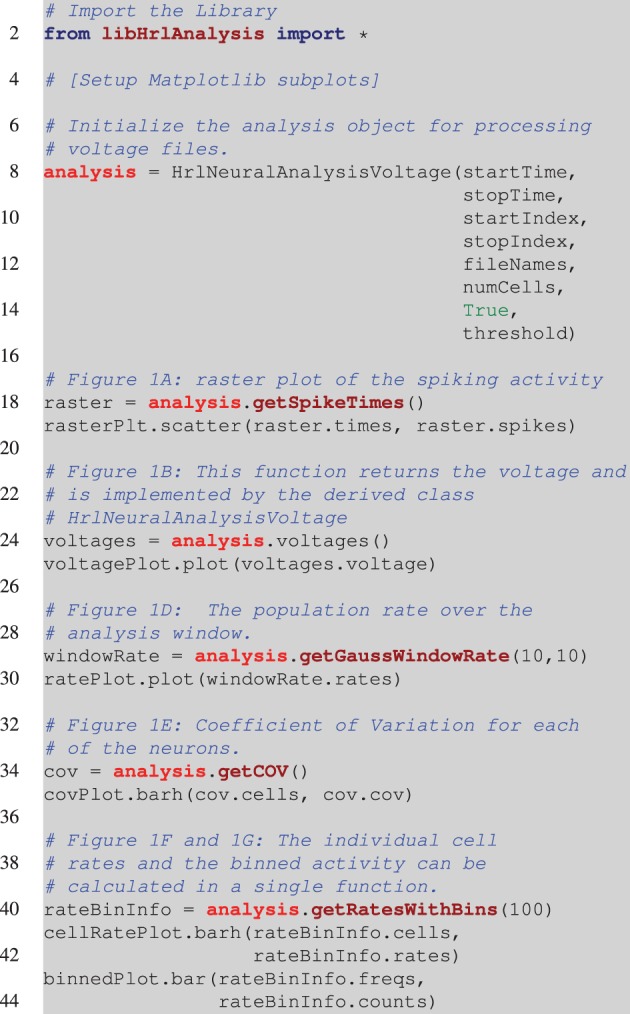
**Pesudocode for creating Figure 1**. This is incomplete and assumes that the appropriate Matplotlib axes are setup on Line 4. The analysis object is instantiated on Line 8. This is a voltage object that is specific to processing HRLSim^™^ voltage files. As arguments the constructor requires, *startTime* and *stopTime*, which define the region of interest within the experiment, the range of neurons defined by the *startIndex* and *stopIndex* variables, a vector with the file paths containing the raw voltage data as the *fileNames* variable, the number of cells in this voltage file, *numCells* (this is because of the data format), a boolean to tell the analysis if the voltages should be converted into spikes, and finally the membrane voltage threshold for extracting spikes, *threshold*. The remaining lines of code perform the analysis and create the individual plots. Note that Figure 1C is created using a separate analysis object that extracts information about a different cell population but is exactly the same as Line 18.

Each of the different analysis functions—Lines 18, 24, 34, and 40—return pointers to specific structures. In python the different data members of these structures are seen as lists and can be seamlessly plotted with Matplotlib—Lines 19, 25, 30, 35, 41, and 43. This example highlights the simplicity of using HRLAnalysis^™^ but hides the complexity of the design; in particular that these objects are implemented and instantiated in C++.

### 2.2. Design

The hybrid language design of HRLAnalysis^™^ is facilitated by the Boost Python package which provides the logic required to interface C++ with Python. Boost was chosen over SWIG^4^ for its ease of use and additional features. These include namespace support, automatic support for return by pointer and base-class reference, templated type-conversions, and support for multiple source files.

A data-centric object-oriented design (OOD) pattern was selected for its balance of performance and extensibility, as well as for its amenability to interfacing with Python. With this, structures that organize the spike information in ways that are optimal to the analysis can be developed by combining the data with the methods. This allows for algorithmic and compiler optimizations—since the form of the data structures are known and directly accessible—as opposed to relying on decoupled data and access functions where the formats may not be consistent. Beyond the performance of data encapsulation, is the extensibility OOD provides through object inheritance. Users can extend the main library, described below, by adding or modifying functionality to fit their particular needs in derived class objects.

The rationale for employing a Python interface rather than MATLAB is similar. The object oriented aspects of Python make it far easier to reuse than MATLAB (Spacek et al., 2009). In addition, this provides flexibility that simplifies the management of large-scale projects. Conversely, MATLAB functions are written in individual files making it difficult to make major modifications to an existing codebase (Ince et al., 2009). Finally, since Python is open source there is potentially a larger available user base—all that is required is a compatible operating system as opposed to a commercial license.

#### 2.2.1. Core classes

**Figure 2:**
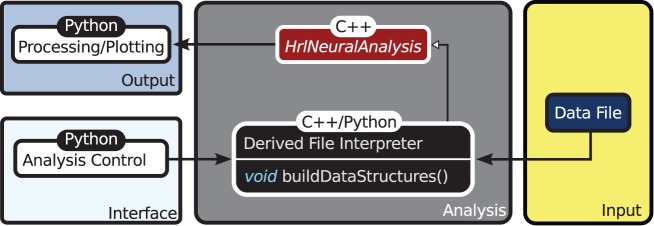
**Context diagram**. The *HrlNeuralAnalysis* base class is the core of HRLAnalysis^™^. This is written in C++ and contains the data structures and analysis methods. Derived classes are created in either C++ or Python to handle the conversion of the data file input into the two core structures. These classes are responsible for implementing the *buildDataStructures* virtual function. The access control and visualization is then provided through the Python interface.

The organization of HRLAnalysis^™^ is illustrated in the context diagram of Figure 2. The abstract base class *HrlNeuralAnalysis*, written in C++, is the core of the analysis context. The data structures—described in section 2.2.2—and core analysis functions—described in section 2.2.3—are contained within this class.

Specialized child objects that derive from the base class are then defined to process different spike data formats—this can be from files, live recordings or running simulations. These are only responsible for implementing the virtual function *buildDataStructures* and constructing the internal data structures, described below. In addition, these derived classes can extend the core functionality by adding additional analysis functions.

The execution of these core functions is controlled from the Python interface. As an example, the provided derived class that handles the HRLSim^™^ data format would be instantiated in Python using

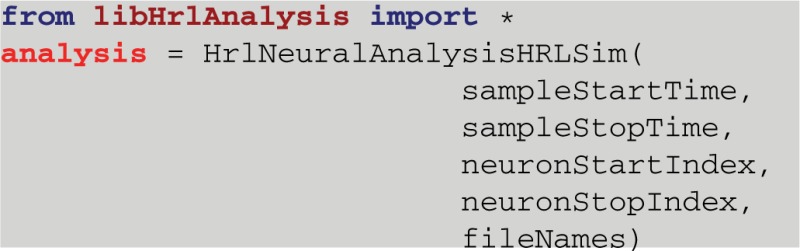



The constructor reads in the bounds of the analysis window, *sampleStartTime* and *sampleStopTime*, which define the region of interest within the experiment. The range of neuron indices is then defined by the *neuronStartIndex* and *neuronStopIndex* variables. Finally, a vector, composed of the file paths containing the spikes, is passed in as the *fileNames* variable.

At this point *analysis* is not completely initialized since *buildDataStructures* has not been called—it is bad practice to call a virtual function from a constructor (Sutter and Alexandrescu, 2004). The user does have the option of directly calling an analysis function since the core library will check if the internal structures have been constructed and *buildDataStructures* will be called if not. Alternatively, *buildDataStructures* can be called directly using

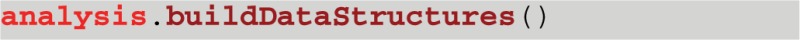



The resulting data structures are described below.

#### 2.2.2. Data structures

**Figure 3:**
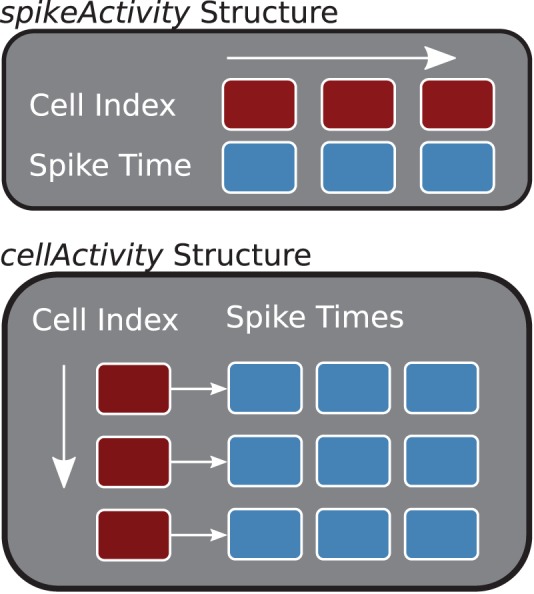
**Core Data Structures (C++)**. The *spikeActivity*, top, stores sorted pairs of spikes times and cell indexes in a vector. The *cellActivity* structure is two-dimensional vector containing spike times for each of the cells.

The two main storage containers constructed in the *buildDataStructures* function, *cellActivity* and *spikeActivity*, organize the spike information by cell or time (see Figure 3). Internally, *spikeActivity* is a Standard Template Library (STL) vector (Stepanov and Lee, 1995), of STL pairs, containing spike time and cell index, ordered by spike times. Similarly, the *cellActivity* structure is implemented with a two-dimensional STL vector, where the first dimension corresponds to the neuron and the second contains the spike times for that neuron. The core analysis methods, described below, will utilize the structure that offers the best performance.

Placing the information in redundant structures sacrifices memory but provides the highest performance when a user has the need for the associated analysis methods—it is more efficient computationally to build these with the raw data than convert from one to the other. This is the case in the example derived classes provided with the suite. However, there is no requirement that a derived class build both structures. In addition, future releases of the library will give the user the option of using just-in-time construction of only the structure that is required when they call an analysis function.

With this design the base object encapsulates both the data and the analysis methods—sacrificing generality for optimization and consistency. This runs counter to the current trends in computational neuroscience where most, including the authors, are pushing for more collaborative projects and standardized data formats. The decision for internal structures was based on the need for performance. However, as we demonstrate below this does not impede interoperability.

#### 2.2.3. Analysis functions

HRLAnalysis^™^ provides a useful set of basic functions for spiking probability and statistics. This particular collection of methods was selected based on the initial needs of the authors for dealing with large amounts of simulated data. They therefore do not cover the full breadth of possible spike-train analysis. Additional functions are constantly being developed and, once mature, will be included in future releases. The currently supported functions, listed in Table 1, are briefly described below. For more information about the implementation of these refer to the references or the available codebase.

**Table 1:** **HRLAnalysis^™^ spike analysis functions currently available**.

**Function name**	**Description**	**Structure**	**References**
*getBursting*	Identify periods of bursting for individual cells using Poisson surprise	Cell activity	Hanes et al., 1995
*getPairSynchrony*	Bivariate spike dissimilarity between two cells using the SPIKE-distance method	Cell activity	Kreuz et al., 2013
*getPopulationSynchrony*	Bivariate spike dissimilarity for the entire population using the SPIKE-distance method	Cell activity	Kreuz et al., 2013
*getCOV*	Calculate the coefficient of variation for cells that had more than 10 spikes	Cell activity	Dayan et al., 2001
*getCellRates*	Calculate the individual cell spike count averages	Cell activity	
*getRateBins*	Bin the number of cells based on average spike counts	Cell activity	
*getRatesWithBins*	Combines *getCellRates* and *getRateBins*	Cell activity	
*getGaussWindowRate*	Approximate the fire rate of the population using a Gaussian window	Spike activity	Dayan et al., 2001
*getWindowRate*	Approximate the fire rate of the population using a rectangular window	Spike activity	Dayan et al., 2001
*getSpikeTimes*	Create separate vectors of cell index and spike time	Spike activity	

The spike-count rate, *r*, of a single neuron is a simple way to quantify the average activity over a window of time. Despite the loss of temporal information this metric is useful in characterizing the response of a neuron to a stimulus (Dayan et al., 2001). These can be used to find the tuning curves of neurons and their distribution within a population can reveal stimulus selectivity or redundancy in the network. In HRLAnalysis^™^ the spike-count rate for each of the neurons is found using *getCellRates*, the spike-count histogram is returned by *getRateBins*, and both can be computed using *getRatesWithBins*—the combined method reduces redundant computations.

The loss of temporal resolution can be partial restored by approximating the firing rate, *r*(*t*), of a single neuron or a whole population. This can give a better representation of the stimulus tuning. HRLAnalysis^™^ provides two methods for approximating the firing rate. The *getWindowRate* method counts spikes along a rectangular window that is slid over the spike trains; the window size and step size are defined by the user. In addition, these can also be filtered using a Gaussian window function that smooths the response by weighting the influence of the cells before and after the current time. This is provided by the *getGaussWindowRate*.

Although important, these rate codes can fail to capture important spike response variability or information about disparate cells within a population. For a single neuron this variability can be captured by the inter-spike interval coefficient of variation (COV)—assuming a stationary rate. The *getCOV* function performs this calculation. The COV is useful for quickly identifying neurons with irregular spiking activity over the period of analysis. In addition, this is a necessary condition for identifying Poisson firing (Dayan et al., 2001).

The COV reduces neural variability to a single number but often the quality of that variability is important. One example is in capturing the bursting of a spike train. Bursting can be an indication of a pathological condition, such as in Parkinson's disease (Rubin et al., 2012). It can also be an important indicator of neuronal modulation (Hanes et al., 1995). The *getBursting* function is used to find the regions of bursting for each of the neurons in a population. This can be used to explore metrics such as the synchronization of bursting or the overall burst rate of a population.

Along the same lines, the correlated firing of cells within a population can both indicate a pathological condition (Walters and Bergstrom, 2010), as well as reveal details of stimulus encoding (de la Rocha et al., 2007). HRLAnalysis^™^ provides both average measures of spike-train synchrony as well as instantaneous synchrony between cells over the sample interval using the SPIKE-distance method of Kreuz et al. (2013). The instantaneous dissimilarity between two cells is found using *getPairSynchrony* and can be found for the entire population using *getPopulationSynchrony*. The results of these can be used to find a single distance metric using either *calcSPIKEDistance* or *calcSPIKEDistance* to find the bivariate SPIKE-distance by numerical integration using Simpson's rule or taking the average of the dissimilarity profiles, respectively. This a useful metric for effectively obtaining a comparable representation of the overall synchrony between spike trains.

It is important to note that all of these functions are implemented in the *HrlNeuralAnalysis* base class and directly accessible to the Python functions. Once the spike data has been extracted, the use of these methods is relatively simple. Returning to the example above, to calculate the COV for each of the cells the user would call 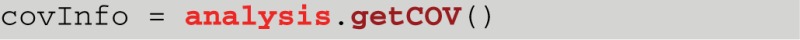


The resulting object contains a vector—seen as a list in Python—of the COVs for each of the cells. All of the analysis functions return similar objects that encapsulate the results. The motivations for this design are described below and for a complete reference refer to the available codebase.

#### 2.2.4. Implementation

Algorithmically, the development of the analysis functions was aided by their coupling to the data structures. As mentioned above, those structures are constructed from STL vectors as opposed to creating custom array based storage which may have resulted in increased performance. However, not only would that have made interfacing with Python more complicated, but it would have made the analysis methods more difficult to design and test. In this case correctness was valued over performance.

STL vectors were also used exclusively for local storage in the analysis functions. To increase the performance of these methods memory was reserved before filling the vectors and *push_back* was the only insertion method, resulting in a constant time insertion cost. There were some instances where the size of the vector was unknown *a priori*—such as during burst analysis. For these cases, the use of *push_back* still results in amortized constant time cost (Sutter and Alexandrescu, 2004). STL containers were favored since these are already highly optimized and, based on our previous experience, hand-tuned structures likely would not have resulted in much, if any, performance benefit (Minkovich et al., 2013). Finally, for the same reasons, built-in STL algorithms were used wherever possible (e.g., *sort* and *accumulate*).

Another design consideration was the choice of control structures. All of the analysis functions require iterating over one of the data objects. In C++ this can be accomplished by directly indexing into the container memory and looping over the length of that by incrementing the index. A more portable way of performing this, is through the use of iterators. These are specialized pointers to locations within the structure that can be used to access the data. On the one hand employing iterators makes for more generic and arguably safer code. On the other, there are some instances where this unnecessarily hides details from the compiler that could otherwise be optimized (Sutter and Alexandrescu, 2004). Furthermore, both of these methods lack bounds checking—meaning there is nothing stopping the code from accessing memory beyond that allocated to the structure. The use of *BOOST_FOREACH*—whose underlying implementation employs iterators—removes many of the pitfalls of iterators and leads to much more readable code. Unfortunately, there are some compilers where this can result in a slight performance drop. During initial testing, however, with the core data structures, the performance of *BOOST_FOREACH* was almost identical to using either iterator or indexed based loops. Because of this, its use was preferred during development when the algorithm lent itself. Another option would have been the use of the C++11 standard range-based *for* method. Unfortunately that was not an option for this version of the library.

Another area of concern are memory leaks resulting from passing objects between C++ and Python. Earlier versions of the library relied on dynamically allocated containers being constructed by the user in Python. These were then passed by reference to the C++ algorithms. This design ensured that the memory was appropriate deallocated but was unfavorable for two reasons. The first was that the library would rely on the Python code to provide the appropriate variable type in the correct position within the parameter list. This made the library difficult to use. The second was that it created extra code that unnecessarily complicated the interface.

To avoid this, all dynamically allocated objects were created with Boost shared pointers^5^. These are class templates that use reference counting to ensure that the object they point to is deleted after all pointers to it are deleted. With these, objects created in C++ can be passed to Python and, when they go out of scope, they are guaranteed to be deleted. This not only made dealing with object ownership between Python and C++ more tractable but also removed container initialization code from the Python interface—greatly improving the readability of the user code.

Although performance was a motivating factor in the design of this library, care was taken to not only follow good programming practices but also produce maintainable and testable code. There are some instances where algorithms could be further optimized but much of that would come at the detriment of readability and extensibility. Because of this, HRLAnalysis^™^ is not fully optimized. However, as the library matures and as bottlenecks within these functions are identified, the algorithms will be further enhanced. In addition, as we demonstrate with the benchmarks below, the library still outperforms comparable spike analysis options.

#### 2.2.5. Extensibility

A unique aspect of this design, and a useful feature, is that the derived classes can be either C++ or Python. This provides users with different levels of programming experience access to the high-performance core. Listing 2 is an incomplete example of deriving from the *HrlNeuralAnalysis* base class with Python. The incoming data in this example is stored by cell indexed spike trains. This layout allows the *cellActivity* structure to be constructed in order, however, the *spikeActivity* structure needs to be sorted when built this way (this is handled by the call to the C++ function *sortSpikeActivity*). Alternatively, only the *cellActivity* structure can be constructed and, if required, the *spikeActivity* structure can be created in C++ using the *buildSpikeActFromCellAct* function. This can result in higher performance during the construction of the internal data structures.

**Listing 2:**
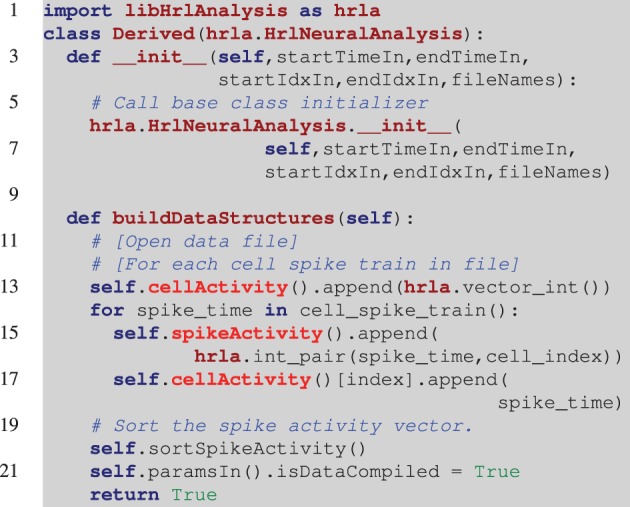
**Example of a derived HRLAnalysis^™^ base object in Python**. In this example the derived constructor prototype matches the base class definition. Notice on line 6 that the base class constructor needs to be called by the derived class. The programmatic loop outlined in line 12 is repeated for each of the spike trains and it is assumed that *cell_spike_train()* on line 14 is provided by that loop. The data structures are incrementally constructed with calls to *append()*. These are C++ data structures, so they map to the C++ vector *push_back()* function. The function call on line 20 sorts the *spikeActivity* structure by spike time. Note that the *spikeAcitivity* structure can be created in C++ by the *HrlNeuralAnalysis* object using the *buildSpikeActFromCellAct* function. This should provide a nominal performance increase over the method presented here.

Extending the base class, whether in Python or C++, does not limit the implementation to spikes. The derived class can be used to process data in different forms as long as the data contains enough information for spikes to be extracted. For example, spike analysis can be done using voltage recordings with the HRLAnalysis^™^ core and a derived class that includes methods specific to voltage information.

Generally, encouraging extensions to a software package can lead to instability. Unit, integration and system tests are included with HRLAnalysis^™^ to combat such software entropy. These are intended to ensure that the core functionality remains stable as users develop their own modules.

### 2.3. Benchmarks

As a way of quantifying the performance of HRLAnalysis^™^, a set of benchmarks comparing it the Python analysis suite NeuroTools^6^ version 0.1.0 were completed. Not all of the analysis functions implemented in HRLAnalysis^™^ have corresponding methods in these packages but a comparable subset was selected. These are listed in Table 2. Only the time to complete the analysis function is measured. This was due to the extended time it took to load the data into NeuroTools which would have drastically altered the results.

**Table 2:** **Benchmark results**.

**Function**	**Cells**	**Analysis time (s)**		**Speedup**
		**NeuroTools**	**HRLAnalysis^™^**	**NeuroTools**
***getSpikeTimes***	1k	0.0112	0.0004	30
*(raster_plot)*	10k	0.1186	0.0050	24
	100k	1.2079	0.0497	24
	1M	12.1409	0.4853	25
	10M	–	4.8941	–
***getRatesWithBins***	1K	0.0019	0.0002	8
*(rate_distribution)*	10K	0.0180	0.0024	7
	100K	0.1744	0.0264	7
	1M	1.7551	0.3711	5
	10M	–	4.1252	–
***getWindowRate***	1K	0.0674	0.0003	209
*(firing_rate)*	10K	0.6615	0.0020	334
	100K	6.5461	0.0182	360
	1M	65.7611	0.1808	364
	10M	–	1.8568	–
***getCOV***	1K	0.0169	0.0012	15
*(cv_isi)*	10K	0.1734	0.0136	13
	100K	1.7176	0.1601	11
	1M	17.1406	1.6327	10
	10M	–	15.8049	–

*These results are for the analysis only. All data structures were initialized before the benchmarks were started. The HRLAnalysis^™^ functions are in bold and the corresponding NeuroTools functions is included below this in parenthesis*.

For test data, Poisson spike trains 10 s long with a target rate of 10 Hz were generated for networks ranging from 1K to 10M cells. However, for NeuroTools the largest network that could be analyzed in memory was 1M cells. The spike trains were saved in binary files with spikes, timing, and size information stored as 32-bit integers. The performance of the analysis methods are dependent on both the number of cells and the number of spikes generated—as both are increased not only does the timing increase but also the file IO. Using independent Poisson generators, rather than a large test network of model neurons, results in a number of spikes that grows somewhat linearly with the number of cells. This was important for exploring the scalability of the library. The number of neurons and spikes generated are included in Table 3.

**Table 3:** **Benchmark results for HRLAnalysis^™^**.

**Cells**	**Number of spikes**	**Time (s)**		
		**Build**	**Methods**	**Total**
1K	99,263	0.0063	0.0021	0.0084
10K	993,727	0.0483	0.0230	0.0713
100K	9,947,156	0.7352	0.2544	0.9896
1M	99,501,298	11.2022	2.6699	13.8721
10M	994,993,689	137.8604	26.6810	164.5414

*The build time is for both reading in the data file and building the data structures. The Methods column contains the total time to run the functions in Table 2. Finally, the last column is the total time to perform the analysis from start to finish*.

Timings for NeuroTools and HRLAnalysis^™^ were computed using the Python Time module. Each benchmark was run three times and the best performance of those was reported. These should be considered approximations, as all are subject to different resolutions based on the underlying implementation. The benchmarks were completed on a server with dual Intel Xeon E5550 2.67 GHz CPUs and 48 GB of memory running CentOS 5.4. Python version 2.6.5 were used.

## 3. Results

### 3.1. Benchmarks

Compared to NeuroTools, HRLAnalysis^™^ is on average 107 times faster. This is a significant performance difference but one that is obscured slightly by the *getWindowRate* results. The speedup for this function is a consequence of the optimized *spikeActivity* structure. It is likely that the performance difference of this function would drop if the conversion to a time based orientation was not required in NeuroTools.

The performance that these benchmarks illustrate is not only important for large neural networks but also for large numbers of small network simulations. The parameter searches described in the conclusion of Thibeault and Srinivasa (2013) are on the order of 1.5 billion simulations and take over 23,000 h of computation time. Roughly half of that is during the analysis using HRLAnalysis^™^. As a conservative estimate, assume that only half of that number was in the actual analysis functions—around 5750 h—and that only a 10 time speedup over NeuroTools is possible. In that case these simulations would have required 80,500 h of compute time—3.5 times longer than with HRLAnalysis^™^. This is an important factor to consider; especially if researchers do not have unlimited access to computing resources and are instead buying time on a compute cluster.

Another important result of these benchmarks is the scaling of the HRLAnalysis^™^ implementation with respect to data size. Table 2 contains the timing results for reading in and initializing the data structures, as well as the time required to run the methods listed in Table 3. Although the analysis functions presented have approximately linear scaling, the file IO and data initialization scaling does not. Despite that non-linear trend, the suite runs faster than real time—less than the 10 s of simulation time—for the cases below 1M cells. For 1M cells the suite completed initialization and all of the methods in under 1.5 times real time. For the 10M cell case, however, the run time is greater than 10.5 times real time.

These benchmarks are meant only as a reference. Although performance is important in this project, we are not suggesting that HRLAnalysis^™^ is a replacement or a competitor to libraries like NeuroTools. It is quite the opposite, we feel these packages complement each other. Both are important tools for neuroscientists and the Python language makes exchanges between the two possible. The only overlapping analysis methods between HRLAnalysis^™^ and NeuroTools are those listed in Table 2.

### 3.2. Interoperability

HRLAnalysis^™^ not only performs well but, through the Python interface, it can be coupled with other analysis libraries. For example, in NeuroTools a *SpikeList* object contains a list of *SpikeTrains*—this is comparable to the *cellActivity* object described here. The constructor for this object takes in a list of tuple pairs of the form *(cell_id,spike_time)*, a list of all of the cell ids, the starting time of the data, and the end time of the data. Converting from HRLAnalysis^™^ into a NeuroTools object can be done in the single compound line of code: 
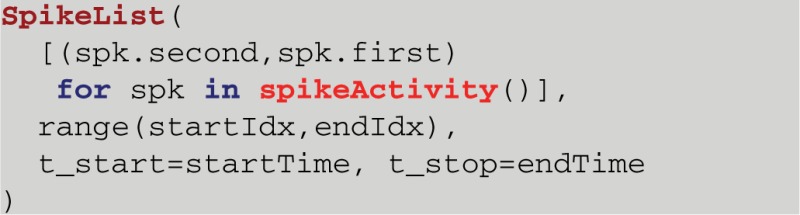


It is this kind of cross tool compatibility that sets Python projects apart from other languages popular in neuroscience. It should be noted that this particular constructor is appropriate only for a small number of cells and spikes. In our benchmarks this conversion took between 1.7 and 1.8 h to complete for the 1M cell case. This was due to the way the *SpikeList* object is constructed in NeuroTools. As the need arises more efficient conversion methods will be implemented and released.

Collaboration is important for data exchange as well as algorithm sharing. The Neo project (Garcia et al., 2014), approaches this by providing a common data object. Developers create interpreters for different open and proprietary file formats that instantiate and return a Neo object. With this, analysis methods can be developed that operate directly on Neo objects while requiring no knowledge of the underlying file format. An example of creating the HRLAnalysis^™^ structures from a Neo object is illustrated in Listing 3. This is incomplete in that there is no error checking and some of the analysis parameters are not filled in (line 27), but it demonstrates one way a Neo object containing spike trains can be incorporated into HRLAnalysis^™^. A complete working example is included with the Python tests in the available codebase.

**Listing 3:**
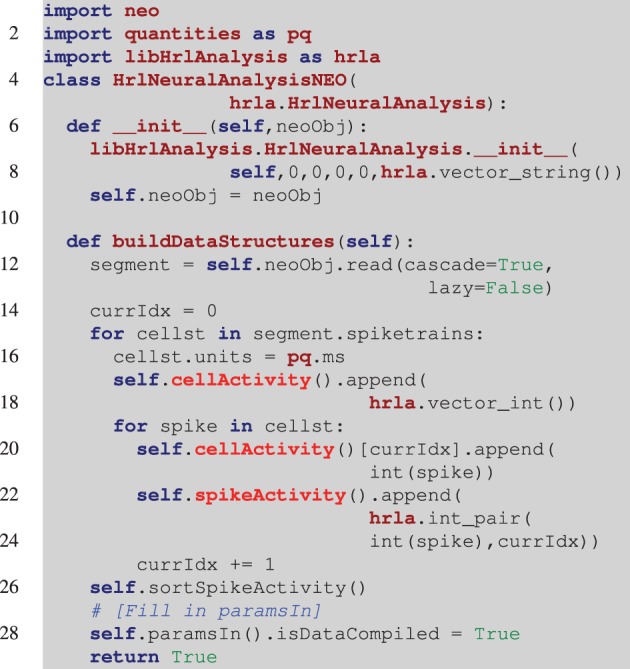
**Example of reading in a Neo object**. Unlike the example in Listing 2, the derived class constructor (line 6), takes a Neo object as an argument. The base class constructor still needs to be called though it is filled with null data initially. In the *buildDataStructures* function (line 11), the spike trains are read from the Neo object and the structures are iteratively constructed. Once again the *spikeActivity* structure is sorted (line 26). Finally, at this point all of the information about the object should be available and line 27 marks the location where the parameters that were set to null in the constructor (line 7), would be set using that information.

## 4. Discussion

### 4.1. Similar projects

There are a number of analysis packages that have been developed for processing simulations and recordings in neuroscience. The International Neuroinformatics Coordinating Facility's software database^7^ contains a large number of projects that span all aspects of neuroscience research. However, we were unable to find a high-performance library for spike analysis that fits our needs.

The aforementioned Python based NeuroTools library focuses minimally on spike train analysis. The broader scope of the project is to reduce the amount of redundant code computational neuroscientists develop and provides functions for designing, simulating and analyzing neural networks. Similarly, the Spykeutils (Pröpper and Obermayer, 2013), library provides methods for analyzing and visualizing spiking information but employs Neo objects as data structures. Neuropy takes a more data-centric approach based on electrophysiological data (Spacek et al., 2009). Information is stored hierarchically based on animal, track and recording. OpenElectrophy deals with large amounts of experimental data by storing it in a MySQL database (Garcia and Fourcaud-Trocmé, 2009). Users can interact with OpenElectrophy through the GUI interface to visualize the raw data as well as perform spike and oscillation detection. The spike detection algorithms are provided but the analysis must be performed by user supplied scripts through the Python interface.

There are similar projects with a focus on high-performance available for MATLAB as well. The FIND package provides both analysis tools for real and simulated single or multi-channel recordings, as well as methods for simulating neurons as point processes (Meier et al., 2008). The Information Breakdown Toolbox is specific to information analysis and takes advantage of MATLAB's MEX interface to speedup computations (Magri et al., 2009). Similarly, the Spike Train Analysis Toolkit (Goldberg et al., 2009), is compatible with both MATLAB and Octave (Eaton et al., 2008), and provides information theoretic analysis of spiking data. This type of analysis is not currently implemented in HRLAnalysis^™^.

### 4.2. Performance, extensibility, and usability

Often when performance is a priority, other desirable software engineering aspects suffer and this project is no different. Although we have aspired to balance performance with extensibility and usability, it is still difficult to achieve all three. As a consequence, this package may have a higher than desired level of specificity.

The complexity of the hybrid language approach may be discouraging to some researchers. In addition, the expectation of a neuroscientist with limited programming experience modifying and adding functions to the core C++ library is unrealistic. However, the option of writing those functions in Python results in a package that not only performs well, but is accessible to users with varying programming experience. It is our intention that as the project attracts more users, these Python prototype functions can be ported into the core library by developers with more experience. This is one of the key benefits of using a hybrid-language design.

### 4.3. Future directions

The large-scale neural simulations that this project was originally intended to support, all rely on parallel processing for efficient computations. Analyzing the results of those using a serial application is somewhat counterintuitive. So the next logical step for HRLAnalysis^™^ is to port the codebase to parallel and distributed systems. Fortunately, having the Python interface can make that transition relatively seamless to the end user. As a first step, we plan to implement the analysis functions in OpenCL^8^. This will provide a standardized interface to a large number of hardware platforms.

Although not discussed here in detail, we have begun implementing network connectivity and synaptic weight analysis tools with HRLAnalysis^™^. These are currently exclusive to file formats output by HRLSim^™^. However, a more generalized set of tools is planned for the future.

## 5. Conclusion

The HRLAnalysis^™^ suite offers both high-performance and usability. More importantly, it complements the existing packages available to researchers today. The capabilities of these different projects and the ability to connect them together through Python is exciting and can only be a benefit to the community. Researchers interested in HRLAnalysis^™^ can access it through the HRL Laboratories Center for Neural and Emergent Systems website^9^.

### Conflict of interest statement

The authors declare that the research was conducted in the absence of any commercial or financial relationships that could be construed as a potential conflict of interest.

## Footnotes

^1^http://www.nih.gov/science/brain/

^2^http://www.boost.org/libs/python/

^3^http://www.matplotlib.org/

^4^http://www.swig.org/

^5^http://www.boost.org/

^6^http://neuralensemble.org/NeuroTools/

^7^http://software.incf.org/

^8^http://www.khronos.org/opencl/

^9^http://www.hrl.com/laboratories/cnes/cnes_main.html
